# Beneficial Effects of Walnuts on Cognition and Brain Health

**DOI:** 10.3390/nu12020550

**Published:** 2020-02-20

**Authors:** Abha Chauhan, Ved Chauhan

**Affiliations:** New York State Institute for Basic Research in Developmental Disabilities, Staten Island, New York, NY 10314, USA; ved.chauhan@opwdd.ny.gov

**Keywords:** Alzheimer’s disease, amyloid beta protein, dementia, depression, oxidative stress, inflammation, mild cognitive impairment, nutrition, walnuts

## Abstract

Oxidative stress and neuroinflammation have important roles in the aging process, mild cognitive impairment (MCI), Alzheimer’s disease (AD), and other brain disorders. Amyloid beta protein (Aβ) is the main component of amyloid plaques in the brains of people with AD. Several studies suggest that Aβ increases the generation of free radicals in neurons, which leads to oxidative damage and cell death. Aβ can also induce neuroinflammation by increasing pro-inflammatory cytokines and enzymes. Walnuts contain several components that have antioxidant and anti-inflammatory effects. Animal and human studies from our and other groups suggest that supplementation with walnuts in the diet may improve cognition and reduce the risk and/or progression of MCI and AD. In the transgenic AD mouse model (AD-tg), we have reported the beneficial effects of a diet with walnuts on memory, learning, motor coordination, anxiety, and locomotor activity. Human clinical trials have also suggested an association of walnut consumption with better cognitive performance and improvement in memory when compared to baseline in adults. Our recent study in AD-tg mice has shown that a walnut-enriched diet significantly improves antioxidant defense and decreases free radicals’ levels, lipid peroxidation, and protein oxidation when compared to a control diet without walnuts. These findings suggest that a diet with walnuts can reduce oxidative stress by decreasing the generation of free radicals and by boosting antioxidant defense, thus resulting in decreased oxidative damage to lipids and proteins. An in vitro study with synthetic Aβ showed that walnut extract can inhibit Aβ fibrillization and solubilize the preformed Aβ fibrils, suggesting an anti-amyloidogenic property of walnuts. Because it takes many years for cognitive impairment and dementia to develop, we suggest that early and long-term dietary supplementation with walnuts may help to maintain cognitive functions and may reduce the risk of developing, or delay the onset and/or slow the progression of, MCI and dementia by decreasing Aβ fibrillization, reducing oxidative damage, increasing antioxidant defense, and decreasing neuroinflammation. Furthermore, several animal and human studies have suggested that walnuts may also decrease the risk or progression of other brain disorders such as Parkinson’s disease, stroke, and depression, as well as of cardiovascular disease and type 2 diabetes. Together, these reports suggest the benefits of a walnut-enriched diet in brain disorders and in other chronic diseases, due to the additive or synergistic effects of walnut components for protection against oxidative stress and inflammation in these diseases.

## 1. Introduction

Dementia is defined by age-related progressive impairment of cognitive function in several domains (memory, learning, judgment, orientation, language, and comprehension), thereby affecting the daily activities of life and social function in elderly people. The prevalence of dementia has been increasing over the years because of an increase in the aging population. In 2015, 47 million people were suffering from dementia worldwide, and 131 million people are estimated to have dementia by 2050 [1]. Dementia occurs mainly in people older than 65 years of age, when comorbidity is also a common occurrence. It is generally preceded by mild cognitive impairment (MCI). The current estimate is that 33% of elderly people die with dementia. The annual cost of dementia care globally is roughly $818 billion.

Alzheimer’s disease (AD) is a devastating neurodegenerative disorder that gradually leads to memory loss and decline of cognitive functions over a time period of 5–20 years. Although there are many types of dementia, AD ranks as the most common cause of dementia among elderly people, and it accounts for over 60% of dementia cases. Vascular dementia is the second most common dementia, followed by dementia with Lewy bodies. AD affects about 25 million people worldwide, including 5.5 million people in the United States. A total of 10% of the population over 65 years of age, and nearly 50% of people over 85 years of age, suffer from AD. The neuropathological hallmarks of AD include neuronal loss and progressive accumulation of fibrillar amyloid beta protein (Aβ) as amyloid plaques, and of paired helical filaments as neurofibrillary tangles in the brain [2]. In AD, the major amyloid protein is amyloid beta protein (Aβ) of 40 or 42 amino acids, which exists as soluble or fibrillar Aβ. Neuropathological changes in AD occur slowly over decades before the clinical symptoms of dementia are diagnosed.

MCI is considered an intermediate state between healthy aging and early dementia [1]. The prevalence of MCI is 10%–20% of people 65 years of age or older. Although these people can perform daily activities, they are considered at higher risk of developing dementia within 3–10 years. Therefore, this time period provides a potential targeted intervention window to reduce the risk, prevent, or delay the onset and progression of cognitive impairment and dementia. MCI is classified as either amnestic MCI (with impaired memory) or non-amnestic MCI (no effect on memory). About 50% of people with amnestic MCI develop dementia in three years.

About 35% of dementia cases are attributed to modifiable risk factors, which include vascular (cardiovascular disease, hypertension, stroke), metabolic (diabetes, midlife obesity), head trauma, depression, and lifestyle factors (diet quality, alcohol abuse, sleep deprivation). It is estimated that 33% of dementia cases can be delayed or prevented through better management and targeted intervention of these risk factors, particularly hypertension, depression, diabetes, and obesity [1]. The association between diet and health is becoming increasingly clear, with extensive evidence that plant foods rich in flavonoids and phenols are efficient as defensive antioxidants, thus reducing oxidative stress, which is known to contribute to the pathophysiology of many diseases, including neurological disorders, cardiovascular disease (CVD), hypertension, and diabetes. In the following sections, we review evidence that early intervention with a walnut-enriched diet can reduce the risk and/or delay the onset or slow the progression of cognitive decline and dementia because of (a) the elevated oxidative stress and inflammation involved in the aging process and dementia and (b) the antioxidant and anti-inflammatory components of walnuts.

## 2. Oxidative Stress in Aging, MCI, and AD

Oxidative stress is caused by an imbalance of free radicals’ levels and antioxidant defense in the body [3]. Increased levels of free radicals are toxic, and if not removed, they react with lipids, protein, and nucleic acids in the cell and damage cellular functions. As a result, oxidative stress affects membrane properties such as fluidity, enzymes’ activities, ion transport, and cross-linking of proteins. Enhanced oxidative damage eventually leads to cell death. The brain is particularly vulnerable to oxidative stress because it consumes 20% of the total body intake of oxygen (due to its higher energy requirement), and it has limited antioxidant capacity and higher amounts of unsaturated lipids.

Several studies with human and experimental models suggest increased oxidative stress [4,5,6,7] and inflammation [7,8,9,10] to be important features in the aging process and in AD, which can cause neuronal dysfunction and death. Enhanced oxidative damage as evidenced by increased lipid peroxidation, protein oxidation, and DNA oxidation has been demonstrated in the brain, cerebrospinal fluid (CSF), and blood samples of individuals with AD. Increased oxidative damage is also reported in the brains and blood samples of individuals with MCI, and in the CSF of individuals with early signs of dementia [11,12,13]. Several reports suggest that Aβ induces neuronal death by increasing oxidative stress [14]. Aβ generation is also increased because of oxidative stress, which then causes more oxidative damage.

## 3. Inflammation in Aging, MCI, and AD

Several studies suggest that neuroinflammation cascades mediated by activated microglia cells, which release proinflammatory cytokines, have a detrimental role in AD [7,8,9,10]. Higher numbers of activated microglia and astrocytes, and elevated levels of inflammatory cytokines, namely, interleukin (IL)-6, IL-1β, and tumor necrosis factor-α (TNF-α), have been reported in aging brains and AD brains. Aβ has also been reported to activate microglia cells, which leads to enhanced production of proinflammatory cytokines (IL-1β, IL-6, and TNF-α) and stimulation of proinflammatory enzymes, e.g., inducible nitric oxide synthase (iNOS), resulting in enhanced NO (nitric oxide) production [15,16]. In AD, the expression of cyclooxygenase (COX-2, induced by proinflammatory mediators) is also upregulated [17], which results in increased production of inflammatory prostaglandins (PGs), especially PGE2, in the brain [18]. Increase in COX activity and PGE2 is also reported in aging brains. Because the PG synthesis pathway is a major source of reactive oxygen species (ROS) in the brain, inflammation may also be partly responsible for elevated oxidative stress in aging and AD. Several studies have suggested the association of chronic inflammation with other diseases, including CVD, diabetes, depression, Parkinson’s disease (PD), and hypertension.

## 4. Antioxidant and Anti-Inflammatory Components of Walnuts: Cumulative Effects

Several lines of evidence suggest that walnuts (*Juglans regia* L.) may reduce the risk of age-related diseases because of the additive or synergistic effects of its components with antioxidant and anti-inflammatory effects. Walnuts have a high content (3.68 mmol/oz) of antioxidants, including flavonoids, phenolic acid (ellagic acid), melatonin, folate, gamma tocopherol (vitamin E), selenium, juglone, and proanthocyanidins [19,20,21,22,23]. In addition, walnuts contain a high amount of n-3 α-linolenic acid (ALA), a plant-based omega-3 fatty acid that has a highly potent anti-inflammatory effect [23,24,25,26]. Walnuts also provide protein (4 g/oz), fiber (2 g/oz), phosphorus (10% daily value), and magnesium (11% daily value).

Of 1113 different food items that were tested for their antioxidant contents, walnuts were ranked second place [21]. Among dry fruits, walnuts have the best antioxidant efficacy, as indicated by the fact that walnuts have the highest phenolic content, followed by almonds and cashew nuts and then raisins [27]. Another report indicated that 50 g of walnuts have significantly more phenolic content compared to an 8-oz glass of apple juice, 5-oz glass of red wine, or a milk chocolate bar [19].

Although most nuts contain monounsaturated fats, only walnuts have mainly polyunsaturated fat (13 g of 18 g total fat per 1 oz walnuts), of which the ALA amount is 2.5 g. ALA is the precursor for eicosapentaenoic acid (EPA) and docosahexaenoic acid (DHA), which are known to have anti-inflammatory effects. Studies have shown that ALA inhibits inflammation by downregulating iNOS (thus inhibiting NO production), COX-2, and inflammatory cytokines (IL-1β, IL-6, TNF-α) [23,24,25,26].

## 5. Beneficial Effects of a Walnut-Enriched Diet on Cognitive Function

Recent animal and human studies from our and other groups have suggested that long-term dietary supplementation with walnuts may reduce the risk or delay the onset/progression of MCI and AD.

### 5.1. Animal Studies

Aβ is produced by the proteolysis of amyloid precursor protein (APP). APP-transgenic mice (AD-tg) with the APP gene mutation show memory deficit and Aβ deposition in the brain and are considered an animal model of AD. The recommended daily serving of walnuts is 1–1.5 oz, i.e., 28–42 g, which is equivalent to 12–18 walnut halves. We examined the effects of long-term (14 months) dietary supplementation with walnuts (6% or 9% that equates to the recommended 1 oz or 1.5 oz of walnut intake per day in humans) on the memory, learning skills, motor coordination, and anxiety of AD-tg mice [28]. AD-tg mice on a control diet without walnuts showed memory deficit, anxiety-related behavior, and impairment in motor coordination, position discrimination learning ability and spatial learning ability in comparison to wild-type mice on the same diet. When fed to AD-tg mice, the diets supplemented with walnuts (6% or 9%) showed an improvement in memory, learning skills, motor development, and anxiety-related behavior compared to a control diet without walnuts [28]. The diets for the control and experimental mice were comparable in terms of total calorie intake as well as protein, carbohydrate, and fat contents [28]. In another study with aged rats (19 months old), a diet with 6% walnuts was also reported to improve cognitive and motor performance [29].

### 5.2. Clinical Trials in Humans

In two PREDIMED (Prevención con Dieta Mediterránea) clinical trials from Spain, healthy adult subjects on a Mediterranean diet supplemented with 30 g mixed nuts/day (15 g walnuts, 7.5 g hazelnuts, and 7.5 g almonds) showed better cognitive function compared to the control group on a low-fat diet [30,31], and memory was significantly improved when compared to baseline scores in that group [31]. The participants in these studies were 522 adults (mean age: 74.6 years) [30] or 447 adults (mean age: 66.9 years) [31] at high cardiovascular risk but without any CVD or cognitive impairment. In the first study [30], global cognitive performance was assessed by the Mini-Mental State Examination (MMSE) and Clock Drawing Test (CDT) at the end of clinical trial after 6.5 years of dietary intervention, but baseline evaluation was not done. The second study examined rates of cognitive change with time by comparing scores from six different neuropsychological tests done at baseline and after 4.1 years of nutritional intervention [31].

In another clinical trial with older women, higher long-term intake of nuts (particularly walnuts) was reported to be associated with better cognitive performance [32]. In this study, 15,467 women (70 years of age or older; mean age: 74 years) participated. The difference in cognition scores between women who took five or more servings of nuts per week and women who did not consume nuts was equivalent to two years of cognitive aging. The National Health and Nutrition Examination Study (NHANES) of an adult population in the United States also showed better cognition scores with walnut consumption [33]. This study examined data from two different age groups (20–59 years; 60 years and older). Cognition scores were better with walnut consumption in both age groups.

Another study in 64 young adults examined the effects of short-term (8-week) dietary supplementation with walnuts on cognitive performance [34]. Memory, mood states, verbal reasoning, and non-verbal reasoning were assessed at baseline and at the end of the 8-week nutritional intervention period. There was a significant increase in inferential verbal reasoning in the subjects on the walnut-enriched diet, but there were no significant differences for mood, memory, or non-verbal reasoning. This may be because dietary supplementation of walnuts occurred for only eight weeks in this study.

## 6. Mechanisms of Beneficial Effects of Walnuts on Cognition and Brain Disorders

**Figure 1:**
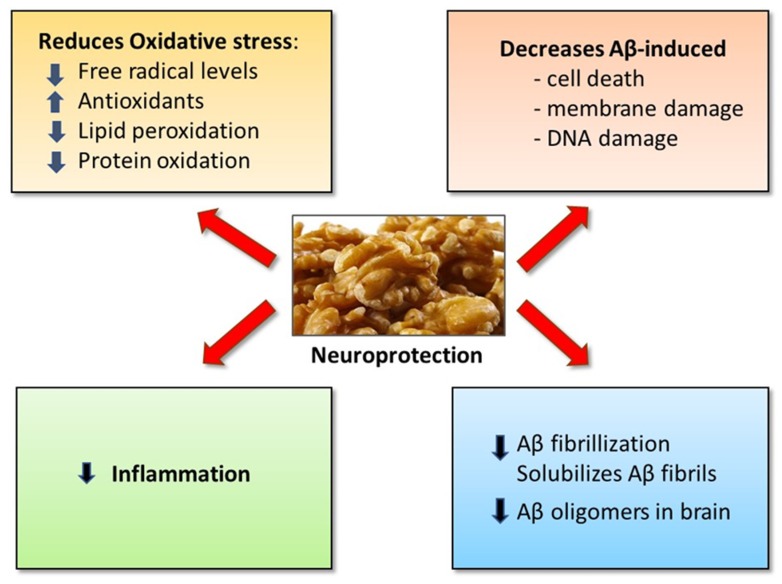
Potential mechanisms for neuroprotective effects of walnuts in reducing the risk of mild cognitive impairment (MCI) and dementia.

Figure 1 elucidates the potential mechanisms by which dietary supplementation of walnuts may reduce the risk, delay the onset and/or slow the progression of age-related cognitive decline, MCI, and AD.

### 6.1. Anti-Amyloidogenic Property of Walnuts: Walnuts Inhibit Aβ Fibrillization and Solubilize Aβ Fibrils

The formation of Aβ fibrils from soluble Aβ is preceded by Aβ oligomerization/aggregation, and it involves change in Aβ conformation from α-helical to β-pleated sheet structure. In a study with synthetic Aβ, walnut extract inhibited Aβ fibrillization and solubilized preformed Aβ fibrils. Here, thioflavin T fluorescence spectroscopy was used to assess the degree of Aβ aggregation/fibrillization, and the morphology of Aβ structure was examined by electron microscopy [35].

### 6.2. Walnuts Decrease Aβ-induced Oxidative Stress and Cell Damage

Many in vitro studies have reported that Aβ exhibits cytotoxic property by increasing ROS levels and inducing oxidative stress [14]. We have reported that walnut extract can protect against Aβ-induced oxidative stress and cell death [36]. In this study with PC12 pheochromocytoma cells, we examined the effects of walnut extract on Aβ-induced cell damage, ROS production, and apoptosis. The intracellular ROS accumulation caused by Aβ treatment to the cells was significantly reduced in the presence of walnut extract when compared to Aβ-treated control cells without walnut extract. Walnut extract also decreased Aβ-mediated cell death (assessed by reduction of MTT (3-(4,5-dimethylthiazol-2-yl)-2,5-diphenyltetrazolium bromide), membrane damage (assayed by lactate dehydrogenase release), and DNA damage (apoptosis) in a dose-dependent manner [36]. These findings suggested that walnut extract can protect against Aβ-induced oxidative damage and associated cell death.

To further understand the mechanisms underlying the beneficial effects of dietary supplementation with walnuts in AD, we recently studied whether short- or long-term supplementation with walnuts in the diet can reduce oxidative damage and/or enhance antioxidant defense in AD-tg mice [37]. AD-tg mice consuming a control diet without walnuts (T0) showed a significant age-dependent increase in ROS levels, lipid peroxidation, and protein oxidation, which was coupled with decreased activities of antioxidant enzymes (superoxide dismutase, catalase) compared to wild-type mice on the control diet. Oxidative stress was observed to be significantly reduced in AD-tg mice on diets with 6% (T6) or 9% walnuts (T9), as shown by lower levels of ROS, decreased lipid peroxidation and protein oxidation, and significant improvement in activities of antioxidant enzymes in these mice compared to control T0 mice. Long-term supplementation with walnuts in the diet for 10 or 15 months was found to be more effective in reducing ROS levels and oxidative damage to lipids and proteins and in improving antioxidant status in comparison with short-term supplementation with walnuts for 5 months in AD-tg mice [37].

In humans, acute consumption of walnuts was reported to increase total antioxidant capacity and to reduce plasma lipid peroxidation [38]. The components of walnuts, such as flavonoids, ellagic acid, gamma tocopherol, and melatonin, are known to have antioxidant and free-radical scavenging properties. Because components of walnuts have strong antioxidant properties, it is possible that the inhibition of Aβ-induced free radical levels by walnuts may be attributed to its effects on the neutralization of ROS.

## 7. Potential Benefits of Walnuts in Other Brain Disorders and Chronic Diseases

Oxidative stress and inflammation play pivotal roles not only in MCI and AD, but also in other brain disorders, such as PD, depression, autism, schizophrenia, bipolar disorder, and several age-related chronic diseases.

### 7.1. Walnuts and Parkinson’s Disease, Depression, Stroke, and Epilepsy

PD is characterized by the progressive loss of dopaminergic neurons and by clinical symptoms, including movement impairment, postural imbalance, tremor, and rigidity. In a mouse model of PD, walnut extract improved symptoms of PD (postural balance, motor coordination, and movement), reduced oxidative stress, and protected neurons [39].

Depression is quite common in people suffering from dementia, and it is considered a risk factor for dementia. More than 20% of people with dementia are also diagnosed with depression, and several other people show symptoms of depression. Cohort studies have shown an association between the number of depressive episodes and the risk of MCI and dementia [40]. In a 28-year follow-up study, symptoms of depression were mainly higher in the 10-year period before the diagnosis of dementia [40,41]. Recently, Arab et al. analyzed data on depression and walnut consumption from a NHANES study in the United States population [42]. They reported significantly fewer and less frequent depressive symptoms among subjects who included nuts, especially walnuts, in their diets as compared to a control group who did not take nuts. The subjects on a walnut-enriched diet showed greater interest in doing things, better concentration, higher energy levels, and less hopelessness. Sanchez-Vilegas et al. [43] also compared data on depression among PREDIMED subjects on a Mediterranean diet enriched with 30 g/day mixed nuts (15 g walnuts, 7.5 g hazelnuts, and 7.5 g almonds) or a control low-fat diet. This study population had men (age: 55–80 years) and women (age: 60–80 years) who were at higher risk of CVD but without any previously documented CVD; 51% of these subjects also had type 2 diabetes. When the entire group was compared, the risk of depression was reduced by 20%–30% (although it was not statistically significant) in the subjects who consumed a nut-enriched Mediterranean diet compared to the control group [43]. However, the risk of depression was significantly lower by 40% among people with diabetes in this group assigned to a Mediterranean diet supplemented with mixed nuts as compared to the control group [43]. The incidence of stroke was also found to be reduced by about 50% in the people who were on a Mediterranean diet supplemented with mixed nuts [44].

Epilepsy affects 50 million people worldwide and is defined by recurrent seizures. In experimentally induced epilepsy in rats, a walnut-enriched diet showed neuroprotective and anticonvulsant effects, and it also reduced mortality [45].

### 7.2. Walnuts and Type 2 Diabetes

It has been estimated that people with type 2 diabetes are more likely to develop dementia compared to non-diabetic individuals. A large cohort study of 83,818 women (age: 34–59 years) showed that dietary supplementation of 1 oz of nuts, such as walnuts, five times or more per week decreased the risk of developing type 2 diabetes [46]. In two other large cohort studies with 58,063 women (age: 52–77 years) in the Nurses’ Health Study (NHS) (1998–2008) and 79,893 women (age: 35–52 years) in NHS II (1999–2009), dietary supplementation of walnuts was associated with a significantly lower risk of type 2 diabetes [47]. The consumption of walnuts also significantly improved endothelial function in adults with type 2 diabetes [48]. The PREDIMED study of long-term intervention with a Mediterranean diet enriched with nuts also reported an association of nuts with a 50% reduction in diabetes [44].

### 7.3. Walnuts and Cardiovascular Diseases

The cardiometabolic risk factors that develop in mid-life (hypertension, obesity, hyperlipidemia) are also considered potential risk factors for cognitive decline and dementia. Several studies have suggested that walnuts in the diet can reduce the risk of heart disease by improving various cardiometabolic risk factors [44,49,50]. A walnut-enriched diet can decrease total and low-density lipoprotein (LDL) cholesterol, increase high-density lipoprotein (HDL) cholesterol, and reduce blood pressure, inflammation, and plaque formation [49,50,51,52,53,54,55]. Due to evidence in support of the benefits of walnuts related to cardiovascular health, the U.S. Food and Drug Administration approved the following health claim for walnuts in 2004: “Supportive but not conclusive research shows that eating 1.5 ounces of walnuts per day, as part of a low saturated fat and low cholesterol diet, and not resulting in increased caloric intake may reduce the risk of coronary heart disease.”

### 7.4. Walnuts and Body Weight

Contrary to expectations, clinical trials, epidemiological studies, and systematic reviews of different studies have shown that walnut consumption in the diet does not contribute to weight gain or hinder weight loss goals as compared to a control diet [49,53,56].

## 8. Conclusions

Oxidative stress and inflammation play important roles in the aging process, MCI, dementia, and many age-related diseases. Walnuts have multiple components with antioxidant and anti-inflammatory effects, which may have additive or synergistic effects in suppressing inflammation and oxidative damage. Our studies have demonstrated that walnuts reduce oxidative stress not only by decreasing free radical levels but also by boosting antioxidant defense, thus reducing oxidative damage to lipids and proteins.

Substantial evidence from animal and human studies suggests that dietary consumption of walnuts (1–2 oz per day) can improve cognitive function and also reduce the risk of other diseases, such as cardiovascular disease, depression, and type 2 diabetes, which are risk factors for the development of dementia. Our studies in AD-tg mice have clearly demonstrated that long-term supplementation with walnuts in the diet can (a) significantly improve memory, learning skills, motor coordination, and anxiety-related behavior and (b) attenuate Aβ-induced oxidative stress by improving the balance between free radicals and antioxidants and associated Aβ-mediated cell death. Together, these reports suggest that early and long-term nutritional intervention with walnuts may have beneficial effects in maintaining cognitive function and protecting against age-related cognitive decline and in reducing the risk, delaying the onset, or slowing the progression of cognitive impairment and dementia in MCI and AD.

In order to clearly understand the mechanisms underlying the anti-amyloidogenic role of walnuts, further studies are warranted to investigate whether dietary walnuts can (a) affect the proteolytic cleavage of APP and inhibit Aβ production and/or (b) increase Aβ degradation by proteases, thus reducing the levels of Aβ in the brain and increasing its clearance.
